# Fergusson’s System of Practical Surgery

**Published:** 1853-05

**Authors:** 


*Fergusson's System of Practical Surgery.* Philadelphia: Blanchard and Lea, 1853.

THAT a new edition of this work has been called for, is of itself sufficient evidence that it is appreciated by the American profession. For sale by our booksellers generally.

